# Scenario simulation analysis of urbanization spatial pattern optimization promoting regional coordinated development: A case study of Guizhou, China

**DOI:** 10.1371/journal.pone.0330821

**Published:** 2025-09-09

**Authors:** Yuzhu Meng, Mingyue Wang, Shu Shang, Zhenlong Hao

**Affiliations:** 1 School of Applied Economics, Guizhou University of Finance and Economics, Guizhou, China; 2 School of Economics and Management, Dalian University of Technology, Liaoning, China; 3 School of Statistics and Mathematics, Zhongnan University of Economics and Law, Wuhan, China; Ain Shams University, EGYPT

## Abstract

This research delves into the optimization of urbanization spatial patterns in Guizhou Province, China. The findings reveal that with regional coordinated development as the central objective and the optimization of urbanization spatial patterns as the strategic focus, a research framework encompassing “temporal and spatial evolution of urbanization - identification and summation of pain points and difficulties - scenario simulation and optimization - strategic goal selection” is utilized to specifically tackle issues pertaining to urbanization spatial patterns. Through the construction of diverse scenarios and rigorous research analysis, an implementation pathway is derived, advocating for “strengthening the central region of Guizhou, fostering urban agglomeration development, reinforcing developmental support points, and promoting regional coordinated development.” This pathway is then applied to the optimization of urbanization spatial patterns in Guizhou. The study proposes the establishment of a “one body with two wings” urbanization spatial pattern, with Guiyang as the primary core and Zunyi, Liupanshui, Bijie, Anshun, Duyun, and Kaili serving as support points for Guizhou’s primary development zone. Furthermore, urban clusters centered on Xingyi in southwestern Guizhou and Tongren in northeastern Guizhou are projected to gradually emerge. The remaining regions will undergo coordinated development, ultimately contributing to the realization of overall regional coordinated development.

## Introduction and literature review

Since the reform and opening-up policy, China has achieved remarkable urbanization progress marked by sustained socioeconomic development and living standard improvements (Ning et al., 2022) [1]. The urbanization rate surged from 17.92% in 1978 to 65.2% in 2023, reflecting an average annual growth of 1.03 percentage points. However, this rapid transition has generated systemic challenges including traffic congestion, inadequate public service provision, environmental pollution, and unplanned urban expansion. (Yu et al., 2022; Zhao et al., 2023; Yang et al., 2023) [2–4]. At the global level, the United Nations 2030 Agenda for Sustainable Development emphasizes spatial pattern optimization as critical for de·veloping sustainable cities and fostering regional coordination. Domestically, China’s 14th Five-Year Plan (2021–2025) explicitly advocates “nurturing urban agglomerations and metropolitan areas through differentiated development strategies to establish a balanced, synergistic, and functionally integrated urbanization spatial framework.” The 20th National Congress further mandates “constructing coordinated urban systems anchored in metropolitan areas and county-led urbanization.” These policy imperatives highlight the urgent need to design rational spatial optimization pathways that simultaneously promote high-quality regional development and address interregional disparities.

The original intent of urbanization spatial pattern development was to optimize resource allocation, foster coordinated regional growth, and enhance living standards. However, empirical evidence indicates that current spatial configurations have inadvertently widened regional disparities. Scholars have examined this paradox through multiple analytical dimensions:

First, regarding spatiotemporal dynamics, China’s urbanization exhibits a distinct gradient distribution characterized by “eastern density vs. western sparsity,” as explained by geographical theories like the “Hu Line” and economic principles such as growth pole theory. By dissecting historical developmental phases—from initial coastal prioritization to subsequent regional coordination efforts and current cluster-driven urbanization—researchers have constructed a three-dimensional evolutionary framework incorporating natural geographic constraints, economic driving forces, and policy responses. This spatial structure demonstrates pronounced path dependence, manifesting in persistent agglomeration effects in southeastern coastal regions while northwestern inland areas remain trapped in low-level equilibrium due to ecological limitations.[5].Secondly, concerning the mechanisms of regional imbalance, existing literature reveals the disequilibrium effects of urbanization spatial patterns across four dimensions: ① Population distribution: Studies show a significant spatial mismatch between urbanization speed and population agglomeration (Chen M et al., 2016) [6], reflected in both the excessive concentration and “big city diseases” in southeastern coastal city clusters and the population decline leading to “shrinking cities” in northeastern and northwestern regions. ② Industrial and employment structure: Western regions face multiple challenges, including lower urbanization rates than central and eastern areas (Yang Peiqing, 2019) [7], lagging market development, insufficient industrial support, and fragmented urbanization systems. This results in significant disparities in employment elasticity across regions and a mismatch between labor skills and industrial upgrading demands. ③ Economic disparities: The gradient opening-up pattern formed during reform and opening—”coastal areas first, inland regions following, border zones lagging”—has created cumulative agglomeration effects, granting coastal regions a “differential advantage” in resource acquisition and population attraction over inland and border areas. ④ Public services: With socioeconomic development, the contradiction between urbanization and basic public service provision has intensified (Zhao et al., 2022) [8]. The “dense east, sparse west” spatial pattern has led to the excessive concentration of high-quality medical and educational resources in developed regions and megacities, creating a vicious cycle of “central city service upgrading vs. peripheral region relative decline,” further widening regional disparities [9–11]. Finally, regarding optimization strategies for urbanization spatial patterns, existing research proposes various approaches from the perspectives of city clusters [12–14] and “Belt and Road” cities [15], integrating the “production-living-ecological” space framework to enhance spatial structure and foster coordinated regional development.

In summary, the original intent of urbanization spatial pattern development was to optimize urban spatial structures while fostering efficient and coordinated regional growth. However, in practice, this process has led to imbalances between regional development and public resource allocation, manifested in three key dimensions: a mismatch between population mobility and urbanization progression, uneven distribution of employment opportunities across regions, and a pronounced widening of economic disparities.While imbalanced urbanization spatial patterns do not inherently cause regional development disparities, they have de facto exacerbated them. Addressing this challenge requires a dual approach: establishing well-functioning market mechanisms complemented by targeted government interventions, and enhancing urban governance capacity. Such measures can guide orderly population flows, promote equitable employment distribution, and narrow interregional economic gaps—ultimately reversing the current unsustainable trajectory.

The optimization of urbanization spatial patterns entails the systematic reconfiguration of essential urban elements through government-market synergy, aiming to establish a rational and efficient spatial framework (Yang et al., 2020) [16] . This process encompasses enhancing urbanization quality while requiring concurrent efforts to narrow wealth disparities, rationalize income distribution, and achieve regional coordination. As an interdisciplinary research frontier, this topic spans economics, geography, and urban planning, yet current scholarship exhibits three critical gaps: firstly, while existing literature thoroughly documents spatial pattern evolution and regional development challenges, most analyses remain confined to diagnostic frameworks rather than prescriptive solutions. Secondly, the methodological discourse lacks systematic integration of qualitative-quantitative approaches, limiting capacity to analyze both macro-regional dynamics and micro-level implementation challenges. Thirdly, few studies provide actionable pathways for operationalizing optimized spatial configurations, particularly regarding institutional mechanisms and policy translation. This paper addresses these gaps through a three-pronged investigation: reconstructing the spatiotemporal evolution mechanisms of urbanization patterns, developing an integrated methodology combining qualitative scenario analysis with quantitative spatial modeling, and proposing implementation frameworks for translating optimized patterns into actionable policies. By focusing on these interrelated questions, the study advances theoretical understanding while providing practical guidance for regional development strategies.

The contributions of this paper are primarily manifested in the following aspects: Firstly, it transcends the methodological limitations of conventional urbanization studies by innovatively integrating scenario analysis with centroid shift analysis. By employing centroid shift analysis to track the migration trajectories of core elements across Guizhou’s counties, we identify five critical factors influencing urbanization pattern optimization. Subsequently, scenario analysis is applied to construct three developmental frameworks (“single-core,” “dual-core,” and “one-body-two-wings”), thereby establishing a robust foundation for spatial pattern optimization. Secondly, addressing the prevalent issues of fragmented indicators and subjective weighting in existing research, we develop a comprehensive five-dimensional evaluation system encompassing innovation capacity, spatial configuration, infrastructure, capital supply, and ecological environment. This system utilizes an enhanced Analytic Hierarchy Process (AHP) for objective weight determination and fuzzy comprehensive evaluation for scoring optimized urbanization spatial patterns. Thirdly, it adds to the empirical evidence for advancing regional coordination development strategies. Taking Guizhou as a case study, this paper utilizes scenario analysis, coupled with Guizhou’s current development status and provincial, prefectural, and autonomous prefecture-level comprehensive plans, to construct an optimized urbanization spatial pattern. With the ultimate goal of achieving regional coordinated development, this paper aims to provide a reference for other mountainous regions globally in pursuing their own regional coordination development strategies.

## Optimization pathways and methods

### Logic of optimization pathways

Urbanization spatial patterns exhibit dynamic trends that evolve with societal development, though their pace and comprehensiveness vary significantly across regions. Current research methodologies tend to emphasize qualitative analyses and empirical summaries, often neglecting the integration of qualitative-quantitative approaches. Moreover, few studies propose actionable optimization pathways with clear operational frameworks.

In summary, this paper proposes an optimization pathway for Guizhou’s urbanization spatial pattern by complementing qualitative and quantitative methods. Taking “optimization of urbanization spatial patterns” as the primary focus and promoting regional coordinated development as the core objective, this paper attempts to construct an optimization pathway through “urbanization spatio-temporal evolution - summary of pain points and challenges - scenario simulation and optimization - strategic goal selection”. ① Urbanization spatial patterns represent the geographical projections of various factors such as economics, population, and employment. Based on scientificity, objectivity, and accessibility, this paper selects data on core factors like economics, population, and employment, and employs the gravity center analysis method to comprehensively study the spatio-temporal evolution characteristics of urbanization spatial patterns and summarize their existing issues, thereby laying a solid foundation for scenario optimization. ② The formation of urbanization spatial patterns is a lengthy and continuous process, during which factors such as population and economy, which are subject to uncertainties and changes, are difficult to predict. Scenario analysis, on the other hand, can effectively address the contradiction between uncertainties in future development and human subjectivity, thus forming a “uncertainty” point of convergence. In conclusion, this provides a basis for optimizing the selection of urbanization spatial patterns. ③ The choice of optimization strategies for urbanization spatial patterns encompasses different scenarios. When considering different scenarios and making corresponding evaluations, it is essential to take into account the sustainability and coordination of regional overall development, making the relatively optimal choice based on the regional development context.

### Optimization methodology

#### Scenario analysis method.

Scenario analysis, a strategic foresight tool originally derived from narrative structures in screenplay development, focuses on constructing plausible future trajectories rather than predicting specific events. This methodology systematically evaluates the implications of alternative pathways through hypotheses, simulations, and impact assessments, making it particularly valuable for addressing complex uncertainties in optimizing Guizhou’s urbanization spatial patterns. These uncertainties span four critical dimensions: policy frameworks, economic conditions, social dynamics, and environmental constraints. By generating multiple future scenarios, scenario analysis enables the simulation of potential structural outcomes and the formulation of adaptive policy options, thereby enhancing both governance resilience and evidence-based decision-making in urbanization planning. Since Herman Kahn, a researcher at RAND Corporation, introduced the concept of “scenarios” into military strategic research in the 1950s and later solidified its methodology in *The Year 2000: A Framework for Speculation on the Next Thirty-Three Years* (1967), scenario analysis has evolved into a critical branch of decision science. From the 1980s onward, it has been widely applied in Western policy domains, including governmental frameworks (Selin, 2006) [17] and corporate strategic choices (Bradfield et al., 2005) [18]. In the 21st century, its applications have expanded to diverse fields such as ecological economics (Lou et al., 2022) [19], transportation planning (Zegras et al., 2006) [20], climate adaptation (Guivarch et al., 2022) [21], and industrial strategy (Culot et al., 2020) [22], demonstrating particular strengths in managing uncertainties within complex systems.

**Fig 1:**
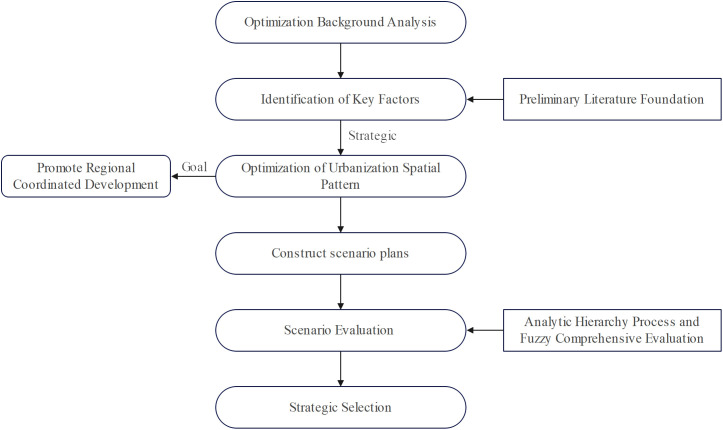
Scenario Analysis Methodology for Optimizing Urbanization Spatial Patterns.

In Guizhou Province, scenario analysis offers three strategic advantages. First, it enables the identification of critical determinants influencing urbanization spatial patterns, including policy orientation, economic growth rates, and population migration trends. Second, building upon these identified factors, the method allows for the simulation of alternative development trajectories by constructing multiple scenarios that model potential spatial pattern transformations under different policy conditions. Third, through comparative evaluation of scenario outcomes, it facilitates rigorous assessment of policy impacts on urbanization patterns, thereby providing an evidence-based foundation for decision-making. The application of scenario analysis is particularly necessary given the multidimensional uncertainties facing Guizhou’s urbanization optimization, which stem from potential fluctuations in policy frameworks, economic conditions, social dynamics, and environmental factors. This methodology effectively addresses these uncertainties by generating robust future projections, highlighting contingency options, and supporting adaptive planning strategies. The implementation framework involves four key steps: establishing macro-level optimization objectives based on Guizhou’s current challenges and developmental vision; systematically identifying and evaluating factors within the urbanization spatial system; designing and analyzing alternative development scenarios; and ultimately selecting the optimal spatial configuration through comparative scenario assessment. This comprehensive approach ensures scientifically-grounded urbanization planning while maintaining the necessary flexibility to accommodate future uncertainties. Based on this, this paper introduces scenario analysis into the optimization of urbanization spatial patterns, with the main steps outlined in Fig 1.

#### Barycentric analysis.

The center-of-gravity analysis method is an approach that involves determining the position of the center of gravity of an object or system for analysis. Specifically, under the action of gravity, an object tends to seek an equilibrium point where the point of action of the resultant force of gravity acting on all parts of the object (i.e., the center of gravity) is in the most stable position. With the development of globalization, economists have gradually utilized the concept of a geographical center of gravity to describe changes in the distribution of world economic activities (Breau et al., 2018) [23]。Drawing on the concept of “center of gravity” from physics, the “center of gravity” in the economic and social context refers to a point in the regional space where the balance of forces in all directions (front, back, left, and right) is maintained. Its primary purpose is to determine the spatial location and movement direction of the regional center of gravity. The formula for this is:


$${\text{ X}}^{\prime }=\frac{\sum_{\text{i}=\text{1}}^{\text{n}}{\text{W}}_{\text{i}}{\text{X}}_{\text{i}}}{\sum_{\text{i}=\text{1}}^{\text{n}}{\text{W}}_{\text{i}}\;}$$ (1)

$${\text{ Y}}^{\prime }=\frac{\sum_{\text{i}=\text{1}}^{\text{n}}{\text{W}}_{\text{i}}{\text{Y}}_{\text{i}}}{\sum_{\text{i}=\text{1}}^{\text{n}}{\text{W}}_{\text{i}}}$$ (2)

In the formula, (${\text{X}}_{\text{i}}$, ${\text{Y}}_{\text{i}}$) represents the centroid coordinates of the secondary unit within the region; ${\text{W}}_{\text{i}}$ represents the attribute value; and the meaning of (${\text{X}}^{\prime }$, ${\text{Y}}^{\prime }$) is determined by ${\text{W}}_{\text{i}}$. This paper selects population, employment, and total economic output as the attribute values of the core elements of the urbanization spatial pattern.

#### Indicator evaluation methodology.

Given the limitations of traditional single evaluation methods, we have adopted an integrated approach combining the Analytic Hierarchy Process (AHP) with fuzzy evaluation to assess the indicators for selecting urbanization development patterns. This approach not only ensures the hierarchical and systematic nature of the evaluation but also effectively addresses the uncertainties and fuzziness inherent in the evaluation process. AHP can intuitively reflect the hierarchical relationships among evaluation criteria and ensure the rationality of weight allocation through consistency checks, thereby enhancing the credibility of the evaluation results. However, its subjective nature means that weight allocation relies on expert judgment, which may be influenced by individual preferences. The fuzzy evaluation method, based on fuzzy mathematics theory, effectively handles the fuzziness and uncertainties in the evaluation process. By transforming qualitative evaluations into quantitative assessments through membership functions, it achieves greater precision in evaluation. Its strengths lie in its flexibility and adaptability, enabling it to process ambiguous information in complex systems and provide evaluation results that better reflect real-world conditions. Therefore, this paper combines AHP with fuzzy evaluation, leveraging the advantages of AHP in weight allocation and the strengths of fuzzy evaluation in handling ambiguous information. The two methods complement each other, collectively improving the comprehensiveness and accuracy of the evaluation.

### Data source and study area

**Fig 2:**
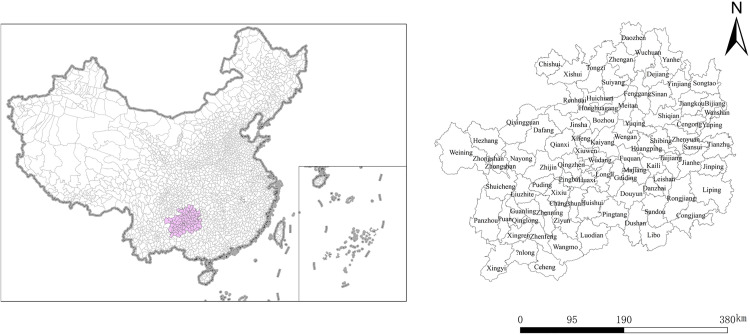
Study area. Note: This map was created based on the standard map with approval number GS(2024)0650 downloaded from the Map World Service Center (https://cloudcenter.tianditu.gov.cn/dataSource), of the National Geographic Information Public Service Platform, and no modifications were made to the base map. When reprinting or quoting this content, it is necessary to clearly indicate “Reprinted from (or cited from) Tiandi Map (https://www.tianditu.gov.cn)”.

The data for this study were sourced from various statistical yearbooks, including the “China County Statistical Yearbook,” “China Regional Economic Statistical Yearbook,” “Guizhou Statistical Yearbook,” “Guiyang Statistical Yearbook,” “Zunyi Statistical Yearbook,” as well as statistical bulletins from municipal, autonomous prefecture, and county-level statistical bureaus across Guizhou Province. Geographic information data were obtained from the 1:4,000,000 database of the National Geomatics Center of China. Guizhou Province, located in the southwest of China, encompasses a total area of 176,000 square kilometers, characterized by a high northwest and low southeast terrain, forming a unique geomorphological pattern known as “eight mountains, one water, one field.” Its specific location within China is illustrated in Fig 2.

## Characteristics and problems of spatial pattern evolution of urbanization in Guizhou Province

### Evolution characteristics of the spatial pattern of urbanization

#### Spatial and temporal evolution of core element gravity.

**Fig 3:**
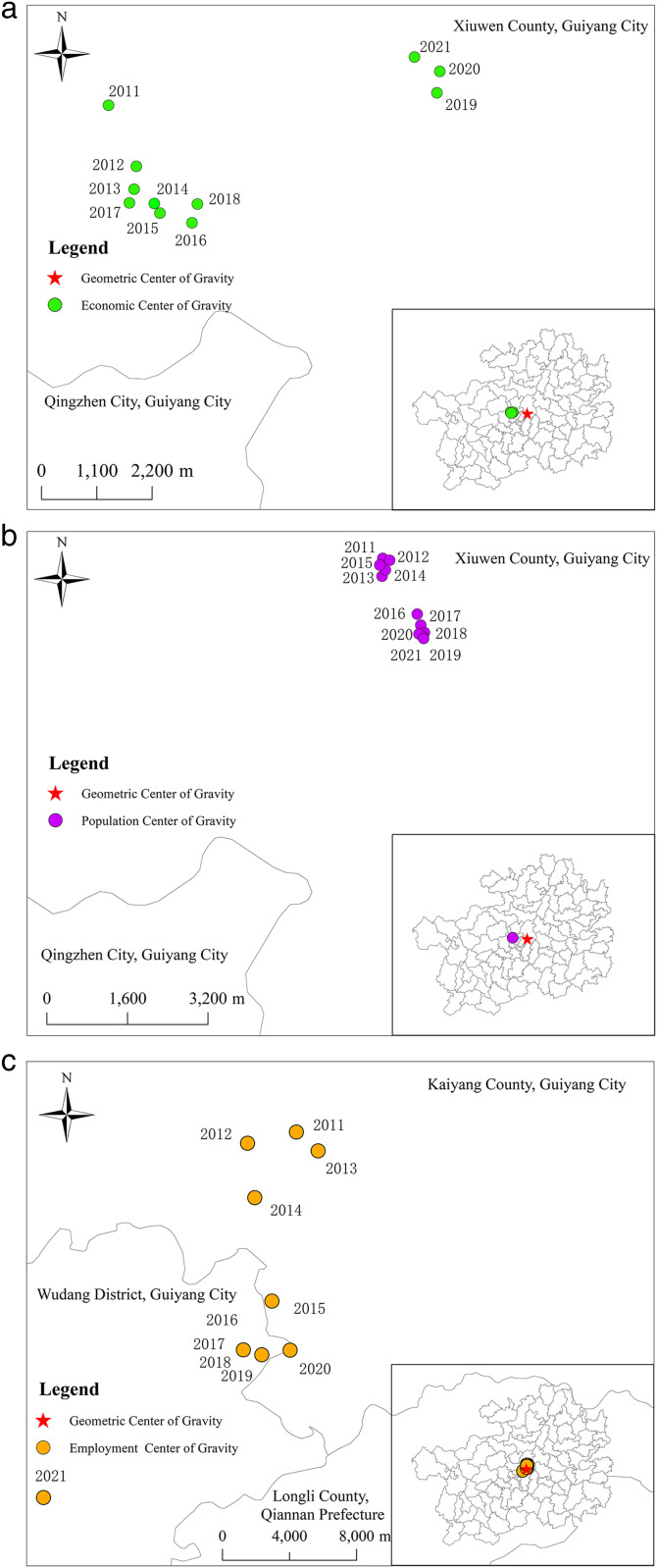
Spatial evolution of population, economy and employment gravity center in Guizhou Province from 2011 to 2021. Note: This map was created based on the standard map with approval number GS(2024)0650 downloaded from the Map World Service Center (https://cloudcenter.tianditu.gov.cn/dataSource), of the National Geographic Information Public Service Platform, and no modifications were made to the base map. When reprinting or quoting this content, it is necessary to clearly indicate "Reprinted from (or cited from) Tiandi Map (https://www.tianditu.gov.cn)".

**Fig 4:**
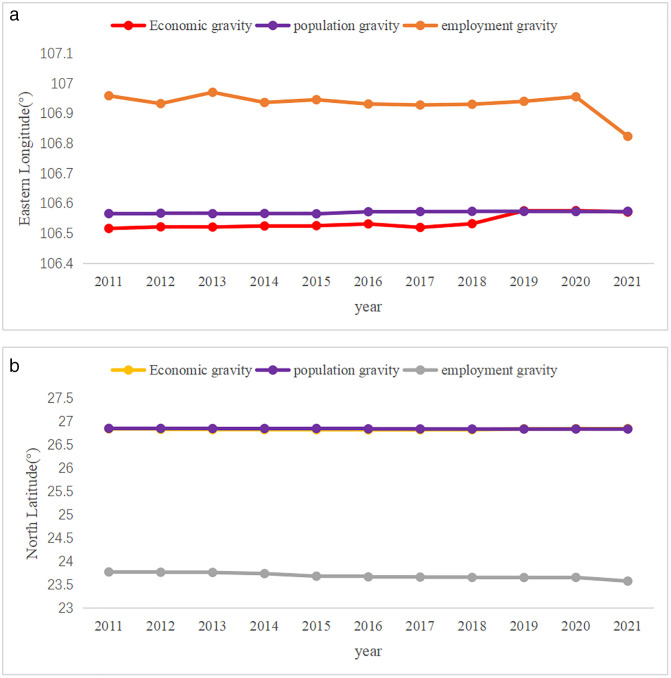
Evolution trend of regional center of gravity in Guizhou Province from 2011 to 2021.

Using municipal-level units as the sub-level statistical units, the centers of economic, population, and employment gravity in Guizhou Province from 2011 to 2021 were calculated using the center of gravity model. The trends in the changes of these centers (ratio of the number of employed in secondary and tertiary industries to the total number of employed) remained relatively stable from 2011 to 2021 (Figs 3 and 4).

The economic center of gravity is generally distributed in the southwest of Xiuwen County, Guiyang City, closely adjacent to Qingzhen City, Guiyang City; the population center of gravity is generally located in the south of Xiuwen County, Guiyang City; and the employment center of gravity is generally located in Kaiyang County, Guiyang City, closely adjacent to Wudang District, Guiyang City, slightly to the east of the economic and population centers of gravity, and it is closer to the geometric center of gravity (107°0′21″E, 26°48′37″N), indicating a more promising employment outlook compared to the other two. The centers of economic, population, and employment are all concentrated in and around Guiyang City, indicating that Guiyang City, with its urban area as the center, is the most concentrated metropolis for labor, capital, and some new technological elements in China. It occupies a dominant position in the spatial pattern of urbanization in Guizhou Province, and its polarization and diffusion effects are gradually increasing, radiating throughout the province and driving the development of more surrounding regions.

In the east-west direction, the economic and population centers of gravity are geographically close and both slightly shifted eastward. After 2019, the trends of these two centers basically coincide, indicating that economic development promotes population aggregation, and vice versa, population aggregation strengthens economic growth. The eastern region of Guizhou Province has relatively rapid economic development, potentially attributed to factors such as its geographical location, resource endowments, or policy support. This rapid economic growth has attracted a significant concentration of population, resulting in a slight eastward shift of the population and economic centers of gravity. This population concentration further stimulates consumption, service industries, and overall industrial development, thereby fostering a virtuous cycle where the economy and population mutually reinforce each other. The employment center of gravity is situated at a 0.5° angle from the economic and population centers of gravity, exhibiting a tendency to shift westward. This may be due to the western region’s advantages in specific industries or sectors, such as agriculture and mineral resource exploitation, which provide numerous employment opportunities. The economic and population centers of gravity possess the capacity to attract labor concentration, while the trends in the economic and employment centers of gravity are more pronounced compared to population changes, indicating that they exert a more significant influence on the spatial pattern of urbanization than population changes do. In the north-south direction, both the population and employment centers of gravity move southward, while the economic center of gravity moves northward. The reason lies in the superior climatic conditions, ecological environment, and transportation facilities in the southern region of Guizhou Province, which have attracted a substantial concentration of population and labor force towards the south. Furthermore, the southern region is actively developing tourism, service industries, and other sectors that have a high demand for labor, thereby promoting the southward shift of the employment center of gravity. Despite the southward movement of the population and employment centers of gravity, the economic center of gravity is shifting northward. This could be attributed to the advantages of the northern region in terms of industrial structure, technological innovation, or policy support, which have fueled rapid economic development. Additionally, the northern region may be actively implementing regional development strategies, such as cultivating economic growth poles and promoting industrial upgrading, which contribute to enhancing the economic strength of the northern region. Compared to the east-west direction, it is evident that the maximum north-south gap is only 0.048°, indicating that the degree of coordination and balance in the north-south direction is significantly higher than that in the east-west direction. This reflects that the overall north-south difference in the spatial pattern of urbanization in Guizhou Province is generally smaller than the east-west difference.

a Economic center of gravity
b Population center of gravity
c Focus of employment

a The east-west evolution of economic, demographic and employment centres of gravity
b Economic, demographic, and employment centers of gravity are evolving north-south

#### Core elements of regional development and evolution.

① Evolution of development in the eastern, central, and western regions. Due to variations in geographical conditions, economic development, spatial planning, policy orientations, and other factors, Guizhou Province can be divided into the Central Guizhou Region, Western Guizhou Region, Northwest Guizhou Region, Northern Guizhou Region, Northeastern Guizhou Region, Eastern Guizhou Region, Southeastern Guizhou Region, Southern Guizhou Region, and Southwestern Guizhou Region. In this paper, the Western Guizhou Region, Northwest Guizhou Region, and Southwestern Guizhou Region are collectively referred to as Western Guizhou; the Northern Guizhou Region, Central Guizhou Region, and Southern Guizhou Region are collectively referred to as Central Guizhou; and the Eastern Guizhou Region, Northeastern Guizhou Region, and Southeastern Guizhou Region are collectively referred to as Eastern Guizhou. The specific administrative divisions are shown in Table 1.

**Table 1:** Regional division of Guizhou.

Region Name	Number of Administrative	Regions Area
Western guizhou	24	All regions of Liupanshui City, all regions of Bijie City except Jinsha County, the entire Qianxinan Prefecture, and Guanling County, Ziyun County, Zhenning County, Xixiu District, and Puding County of Anshun City.
Central Guizhou	37	The entire Guiyang City, Pingba District of Anshun City, the entire Qiannan Prefecture, all regions of Zunyi City except Wuchuan County, and Jinsha County of Bijie City.
Eastern Guizhou	27	The entire Tongren City, Wuchuan County of Zunyi City, and the entire Qiandongnan Prefecture.

The central region of Guizhou, a pivotal pillar supporting the province’s economic and social development, has witnessed a substantial growth in population from 15.0509 million to 16.7536 million between 2011 and 2021, accounting for 43.49% of the provincial total. During this period, the economic aggregate in this region surged from 294.466 billion yuan to 1093.76 billion yuan, contributing 55.86% to the provincial economy. Furthermore, the per capita GDP increased markedly from 19,564.65 yuan to 65,285.07 yuan, exceeding the provincial average of 50,835.62 yuan. In contrast, the eastern region of Guizhou lags significantly behind the central region in both economic and population development. In 2021, the eastern region’s population comprised 19.03% of the provincial total, while its economic aggregate contributed 14.34%, indicating a relatively less developed status. The western region, while not exhibiting a substantial gap with the central region, also lags behind the national average in per capita GDP. With a population share of 37.48% and an economic aggregate contribution of 29.8% in 2021, the western region’s per capita GDP stood at 38,318.10 yuan and 40,422.62 yuan for the eastern and western parts, respectively, both falling short of the national benchmark.

② Evolution of Municipal Districts. As the epicenters of urban development, municipal districts significantly influence the overall economic and social progress of individual cities and, by extension, the entire province. Guizhou encompasses six prefecture-level cities and three autonomous prefectures, within which lie 16 municipal districts. From 2011 to 2021, the total population of these six municipal districts grew from 9.8389 million to 11.3301 million, accounting for 29.4% of the provincial population. Concurrently, the economic aggregate surged from 217.614 billion yuan to 702.816 billion yuan, contributing 35.89% to the provincial economy. This underscores that less than one-third of the population residing in municipal districts contributes over a third of the province’s GDP, albeit falling short of optimal performance. The per capita GDP in these districts escalated from 24,477.73 yuan to 61,651.1 yuan, exceeding the provincial average by 10,815.48 yuan. Additionally, the proportion of employment in the secondary and tertiary sectors within municipal districts rose from 51% in 2011 to 68.3% in 2021, indicative of an increasing trend of rural surplus labor migrating to urban areas for employment, thereby accelerating economic growth.

### Characteristics of the spatial and temporal evolution of urbanization

#### Coordinated development of regional centers of gravity.

The evolution of economic centers of gravity essentially encapsulates the process of regional economic development and mirrors shifts in the polarization and diffusion dynamics of economic activity. The demographic center of gravity reflects the coordinated development of population and the capacity of regions to attract and concentrate population, while the evolution of the employment center of gravity represents the flow and direction of rural surplus labor. Notably, the spatial locations of economic and demographic centers of gravity are particularly proximate, with their trends of change aligning closely from 2019 to 2021. The trajectory of the employment center of gravity deviates towards the economic and demographic centers, signifying a continual enhancement in the congruence of economic growth, population agglomeration, and employment development in Guizhou Province. This, in turn, fosters the development of a more balanced spatial pattern of regional urbanization.

#### Guiyang big city has obvious supporting effect.

After a golden decade of development (2011–2021), Guizhou has successfully cultivated a Type I metropolis in the form of Guiyang City. This achievement has facilitated the concentration of significant labor force and production factors towards Guiyang and its surrounding areas, thereby propelling the development of regional urbanization. Guiyang’s urbanization rate has soared from 69.20% in 2011 to over 80% in 2021, accompanied by a continuous convergence of economic, demographic, and employment centers of gravity. The emergence of Guiyang as a megacity has injected new vitality into the province’s development, forming an economic growth pole that effectively promotes the flow of regional socio-economic factors at lower costs. This, in turn, fosters coordinated development in neighboring regions and gradually extends these benefits to the entire territory of Guizhou.

#### The central region is still the mainstay of Guizhou’s development.

In 2021, the total population of the central region was 16.7536 million, accounting for 43.49% of the province’s total, with a population density of 445.8 persons per km², compared to 208.51 persons per km² in the eastern region and 258.89 persons per km² in the western region. This underscores that the population density in the central region is substantially higher than that of the eastern and western regions. Furthermore, the central region’s GDP also surpasses that of the eastern and western regions, contributing 55.86% of the province’s total GDP in 2021, with a higher per capita GDP than the other two regions. Collectively, these indicators demonstrate that the central region serves as the pillar of development for Guizhou Province, both presently and in the future, and is crucial to optimizing the urban spatial layout.

#### Transportation plays an important role in optimizing the spatial pattern of urbanization.

**Fig 5:**
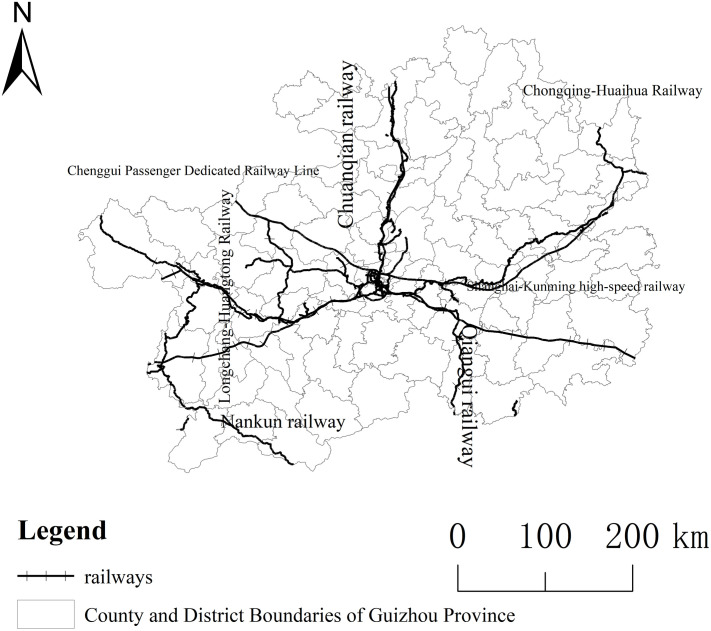
Railway Network Map of Guizhou Province. Note: This map was created based on the standard map with approval number GS(2024)0650 downloaded from the Map World Service Center (https://cloudcenter.tianditu.gov.cn/dataSource), of the National Geographic Information Public Service Platform, and no modifications were made to the base map. When reprinting or quoting this content, it is necessary to clearly indicate "Reprinted from (or cited from) Tiandi Map (https://www.tianditu.gov.cn)" .

As depicted in Fig 5, Guizhou’s railway network comprises major lines such as the Shanghai-Kunming High-Speed Railway, Chengdu-Guiyang High-Speed Railway, Chongqing-Guiyang High-Speed Railway, Guiyang-Guangzhou High-Speed Railway, and the Qian-Gui Railway. Notably, the Shanghai-Kunming High-Speed Railway serves as a pivotal link for east-west transportation in Guizhou, traversing the province from Panzhou in the west to Yuping County in the east, with stops in Pu’an, Guiyang, and Kaili, significantly contributing to the development of Guizhou’s eastern, central, and western regions. The Chengdu-Guiyang High-Speed Railway directly connects Guiyang to Chengdu, fostering economic growth in the northwest of Guizhou. Meanwhile, the Chongqing-Guiyang High-Speed Railway, traversing Zunyi and reaching Chongqing, forms a rapid corridor between the “Guiyang-Gui’an-Anshun” metropolitan area and the Zunyi metropolitan area, while supporting the development of central Guizhou. The Guiyang-Guangzhou High-Speed Railway is a vital transportation route linking Guiyang and Guangzhou, enhancing economic and talent exchanges between Guizhou and Guangdong. Lastly, the Qian-Gui Railway extends north to Guiyang North and south to Liuzhou in Guangxi, serving as the main artery for the movement of people and goods between Guizhou and Guangxi. In conclusion, robust infrastructure, particularly a strong transportation network, is fundamental to a region’s economic development and essential for achieving the goal of optimizing urban spatial patterns.

### Main problems in the spatial pattern of urbanization

#### Economic development is out of step with employment development.

The economic and employment centers exhibit a disparity, with the province’s economic development level declining from central to western and then to eastern regions, whereas the employment level follows a descending trend from central to eastern and then to western regions, indicating an uncoordinated state of development. In 2021, the central region accounted for 43.49% of the province’s total population and 55.86% of its economic aggregate, both exceeding those of the western and eastern regions. Concurrently, the central region remains the primary employment center, with the proportion of secondary and tertiary industry employment reaching 62.85% in 2018, compared to 52.61% in the east and 50.69% in the west. Despite this, the central region’s status as the employment hub persists, yet an ideal level of coordination between economic development and employment distribution has yet to be achieved.

#### The size gap between cities is too large, the overall competitiveness is not strong, and the development is not balanced.

The province comprises a total of 15 cities at various administrative levels, including 6 prefecture-level cities and 9 county-level cities. Among them, there is 1 Type I metropolis, 6 Type II metropolises, 5 medium-sized cities, and 3 Type I small cities (Table 2).

**Table 2:** Classification of City Tiers.

	City Name	Urban Population (in ten thousand)	City Classification
Prefecture-level city	Guiyang City	459.36	Type I big city (300 < 人 < 500口)
Prefecture-level city	Zunyi City	235.75	Type II big city
Prefecture-level city	Liupanshui City	194.77	
Prefecture-level city	Bijie City	129.47	
Prefecture-level city	Anshun City	121.98	
County-level city	Panzhou City	106.94	
County-level city	Xingyi City	100.46	
County-level city	Kaili City	71.72	Medium-sized city
County-level city	Renhuai City	65.42	
County-level city	Qingzhen City	64.1	
Prefecture-level city	Tongren City	60.92	
County-level city	Duyun City	52.82	
County-level city	Xingren City	42.27	Type I small city
County-level city	Fuquan City	29.74	
County-level city	Chishui City	24.69	

Note: The population data presented herein are based on the year 2021, specifically referring to the permanent urban population. The classification criteria for city sizes are clearly defined in the “Notice of the State Council on Adjusting the Criteria for the Classification of City Sizes” issued by the State Council ( Cities with an urban permanent resident population of more than 500,000 but less than 1 million are medium-sized cities; cities with an urban permanent resident population of more than 1 million but less than 5 million are large cities, among which those with a population of more than 3 million but less than 5 million are Type I large cities, and those with a population of more than 1 million but less than 3 million are Type II large cities; cities with an urban permanent resident population of more than 5 million but less than 10 million are megacities; cities with an urban permanent resident population of more than 10 million are supercities.).

Among them, Guiyang stands as the sole representative of Type I metropolis, indicating a lack of diversity. In the future, efforts should be directed towards developing Zunyi City into the second Type I metropolis, thereby providing Guiyang with two growth poles to accelerate Guizhou’s socio-economic development. In the Type II metropolises, Xingyi’s population has just surpassed one million, suggesting a relatively weak development foundation. Tongren, among the medium-sized cities, belongs to a lower-tier city, lagging behind other prefecture-level cities in terms of development. Overall, the city hierarchy reveals that Guiyang, as the core of the province and an economic growth pole, promotes the development of surrounding areas. However, it remains uncertain how long this trend can sustain. Furthermore, when confronted with external competition, cities like Chishui, Renhuai, and Zunyi are influenced by the cross-attraction of Chongqing and Guiyang, while regions such as Xingyi and Liupanshui are affected by the cross-attraction of Kunming and Guiyang, which can diminish Guiyang’s radiation scope and influence. Additionally, Xingren, Fuquan, and Chishui require enhancement in their population agglomeration and economic development capabilities.

#### Regional development is not coordinated, and the polarization trend is increasing.

Since the 21st century, the relatively balanced development pattern in Guizhou Province has been disrupted, with an increasingly strengthened trend towards a single growth pole. Starting from individual cities, the disparities between them have continued to widen. Taking Guiyang and Zunyi, the top two cities in Guizhou, as examples, from 2011 to 2021, the total population growth rate in Guiyang’s urban area was 44.34%, while that in Zunyi’s urban area was only 15.2%. In 2021, the GDPs of Guiyang and Zunyi were 3.6 times and 3.1 times, respectively, that of 2011. From a regional perspective, labor force and other factors of production for socio-economic development have gradually concentrated in cities near the Shanghai-Kunming High-Speed Railway, Chengdu-Guiyang High-Speed Railway, and Chongqing-Guiyang High-Speed Railway. In terms of broader regions, labor force, economy, and other factors of production have gradually converged towards the Central Guizhou Urban Agglomeration.

#### The transportation facilities are weak, and the economic driving effect is weak.

As depicted in Fig 3, regions connected by the Shanghai-Kunming High-Speed Railway and other major trunk lines have achieved relatively better development. In contrast, regions such as Qianxinan and Tongren lack significant railway trunk lines, leading to increased costs in transportation of both human and material resources, posing challenges to their local economic development. Additionally, the transportation network in Qiannan is relatively sparse, contributing to higher transportation costs. Inadequate transportation is one of the reasons for slower population growth and economic development in these cities, manifested in weak industrial development, poor employment environments, and inadequate basic public services.

## Scenario optimization and strategic choice of urbanization spatial pattern in Guizhou Province

### Optimize background and identify key factors

#### Optimize background analysis.

Through the analysis of Guizhou Province’s economic, population, and employment centers, as well as the current characteristics of its urbanization spatial pattern, traditional construction methodologies propose various spatial layout models, which may lead to the loss of development advantages or the amplification of shortcomings in some cities, resulting in uncertainty in urban development directions. The lack of clarity in urban development positioning can give rise to issues in urban spatial strategic layouts, such as insufficient basic public service facilities. Urban planning undertaken by administrators may conflict with the primary functions of cities. Inappropriate industrial locations can hinder the development of other sectors. Therefore, clarifying the promotion of coordinated regional development is both the objective of optimizing the urbanization spatial pattern and the theme of scenario analysis.

#### Identification of key factors.

Through a thorough analysis of the developmental backdrop and regional coordination objectives of Guizhou Province, and drawing upon the research of scholars in fields such as regional coordination and urban planning, it is found that the primary factors influencing the future urbanization spatial pattern of Guizhou Province include economic aggregate, population size, employment status, innovation-driven capabilities, urban construction funding, public service development, transportation infrastructure construction, and ecological and environmental changes. Within a certain period, changes in economic aggregate, population size, and employment status are relatively stable and can be predicted through econometric models, serving as constant variables. However, due to the subjective influence of factors such as national, provincial, and municipal policies and economic strengths, predicting other variables is more challenging, thus they can only be treated as hypothetical conditions. Nevertheless, these variables hold significant importance in promoting coordinated regional development and rational urbanization layouts.

Innovation serves as the primary driving force for development. In recent years, Guizhou has thoroughly implemented the innovation-driven development strategy, consistently placing innovation at the core of its overall modernization efforts. The province has vigorously pursued the strategy of strengthening the province through distinctive science and technology, vigorously promoting technological innovation and other aspects of innovation, fostering an atmosphere of innovation throughout Guizhou, and comprehensively supporting high-quality economic and social development. Grasping the crucial variable of scientific and technological innovation is a vital means to achieve high-quality urbanization, providing robust technological and talent support for the optimization of urbanization spatial patterns.

Investment in urban construction funds. Firstly, the investment of urban construction funds directly affects the development, scale, and construction quality of cities. Secondly, the allocation of funds facilitates the achievement of regional planning objectives, thereby further fulfilling the functions planned for the city. Thirdly, the investment of funds can impact the implementation of large-scale urban construction projects, which in turn provide a significant number of jobs and economic benefits for the city. Fourthly, as government policies continue to evolve, the channels for obtaining these funds are gradually expanding, leading to more rational and diversified use of the funds.

Public Service Development. Apart from emphasizing scale and economic growth, urbanization development also accords greater importance to aspects such as the level of urban public services (Tahmasbi et al., 2019) [24]. In the 21st century, the construction of public service facilities has emerged as a top priority for government work, encompassing the improvement of urban and rural public facilities, as well as the development of public utilities such as education, culture, and sports. In the process of urbanization, the demand for basic public services of the agricultural transfer population is bound to require the planning and design of the supply of urban basic public services (Chao, 2018) [25]. Consequently, the optimization of urbanization spatial patterns must inevitably consider the enhancement of basic public service supply and the rational location and allocation of these facilities.

Transportation Infrastructure Construction. Firstly, the east-west Shanghai-Kunming High-Speed Railway, the central-northern Sichuan-Guizhou Railway and Chongqing-Guiyang High-Speed Railway, as well as the Guizhou-Guangzhou High-Speed Railway and Guizhou-Guiyang Railway in southern and southwestern Guizhou, significantly enhance the mobility of resources and labor within these regions, laying a transportation foundation for high-quality economic development. Secondly, by leveraging the high accessibility of transportation hubs, it is crucial to develop the hub areas and surrounding regions reasonably, forming suitable transit sites and establishing a transportation network with neighboring cities. Thirdly, as transportation infrastructure serves as the cornerstone of economic development, the government should spare no effort in road construction, thereby fostering a four-level transportation network within the province, connecting cities, counties, towns, and villages. This plays a pivotal role in enhancing transportation efficiency within the province, optimizing the urbanization spatial pattern, and promoting regional coordination.

Ecological and Environmental Changes. With the continuous increase in urbanization rates, there seems to be a neglect of the finiteness and scarcity of resources, leading to elevated urban ecological risks and, in recent years, concomitant degradation of rural ecological environments.

with the rapid development of urbanization, there exist a series of environmental problems, including habitat degradation, biodiversity reduction, heat island effect, water bloom effect, and congestion effect, which cause challenges to human life and do not contribute to sustainable development (Hao et al., 2018; Ochoa et al., 2018) [26,27].But these emerging ecological issues pose severe challenges to the high-quality development of urbanization while also offering a path towards harmonious coexistence between humans and nature. “Lucid waters and lush mountains are invaluable assets,” thus, the optimization of urbanization spatial patterns must be grounded in ecological security, forging a new, unprecedented path that harmonizes human activities with nature and promotes coordinated regional development.

### Scenario construction and analysis

#### Scenario construction.

Through an analysis of the optimization background and key influencing factors in Guizhou Province, combined with the overall planning of Guizhou Province as well as the master plans of its six prefecture-level cities and three autonomous prefectures, we aim to construct an urbanization spatial pattern that aligns with the current development reality and future trends of Guizhou Province. On this basis, integrating the four ambitious goals of Guizhou Province in establishing itself as a national ecological civilization demonstration area, a national comprehensive big data pilot zone, an inland open economy demonstration area, and a pioneering region for rural revitalization in the west, we have further refined the framework for scenario construction.

Scenario construction typically consists of 5–7 steps (De Jouvenel, 2000) [28], with the optimal scenario construction being characterized by reasonable, content-rich, and innovative scenarios that are also more easily accepted. The scenario construction for the urbanization spatial pattern in Guizhou Province is divided into the following steps: Firstly, identify the core elements for constructing the urbanization spatial pattern, namely the current development status, trends, and constant and variable factors influencing the urbanization spatial pattern. Secondly, identify uncertain factors. Combining the development characteristics and external environment of Guizhou Province, three combinations of the most likely uncertain factors are identified (as shown in Table 3). Thirdly, set scenario hypotheses. Based on the combinations of uncertain factors, three scenario hypotheses are set: a single-core urbanization spatial pattern, a multi-core urbanization spatial pattern, and an integrated dual-wing urbanization spatial pattern. Fourthly, simulate processes and methods. Using a combination of quantitative and qualitative analysis, each scenario is simulated through the construction of mathematical models and expert assessments. During the simulation process, the range of variation and potential impact of uncertain factors are fully considered to ensure the accuracy and reliability of the simulation results. Finally, analyze and compare the results. Detailed analysis and comparison of the simulation results for each scenario are conducted to examine the evolution trends and potential issues of the urbanization spatial pattern under different scenarios. At the same time, further optimization and adjustment of the simulation results are made in combination with the development goals and actual situation of Guizhou Province.

**Table 3:** Three Development Scenarios of Urbanization Spatial Patterns Based on the Most Likely Combinations of Uncertainties.

Factors/Scenarios	Mononuclear	Multinuclear	Lamellar
Innovation-driven ability	+	++	+++
Investment in urban construction funds	+	++	+++
Public service construction	+	++	+++
Transportation facilities construction	+	++	+++
Ecological environment change	+	++	+++

Note: “+” indicates limited change in the factor; “++” indicates moderate change in the factor; “+++” indicates significant change in the factor.

#### Scenario analysis.

With the constructed urbanization spatial patterns as the target and promoting regional coordination as the orientation, this study comprehensively considers five uncertain factors and retroactively deduces the paths and strategies for realizing each development scenario from the future perspective.

① “Single-Core” Development Scenario. This development scenario is based on limited factor changes, with a comprehensive consideration and compromise approach to factors other than the five mentioned in the previous table. The Guiyang-Gui’an-Anshun Metropolitan Area serves as the core of Guizhou Province, with Anshun leading the development of Xingyi and Liupanshui; Guiyang guiding Bijie, Kaili, and Duyun; and while Guiyang leads Zunyi, Zunyi concurrently guides the development of Tongren. This forms a single-core, multi-cluster network structure. In parallel with the urbanization development of the prefectural regions, a development chain should be established, where urban areas drive county-level regions, and county-level regions drive towns, ultimately forming a comprehensive and all-encompassing development network for Guizhou Province, aimed at promoting regional coordination.

The realization of this scenario entails four primary strategies: Firstly, enhancing the construction of transportation trunk roads in adjacent regions to foster socioeconomic exchanges and enable better-developed areas to drive and elevate the economic strength of neighboring regions. Secondly, reasonably increasing expenditures on innovative research, enhancing innovation capabilities, and cultivating talents for high-tech industries whenever conditions permit. Thirdly, prioritizing ecological environment construction, minimizing disturbances to the natural environment, and reasonably establishing parks in urban areas to create an ecological Guizhou. Lastly, despite limited resources, intensifying investments in basic public service facilities and striving to achieve regionally coordinated development in Guizhou Province. However, the “single-core” development scenario may lead to issues such as excessive concentration of resources, traffic congestion, strained public service facilities, and excessive pressure on the ecological environment, which in turn could impact the development of other regions. Additionally, it may increase the difficulty and cost of urban management, further exacerbating regional imbalances.

② “Multi-Core” Development Scenario. This development scenario is based on moderate variations in various factors, implying an enhanced innovation-driven capacity compared to the “Single-Core” scenario, moderate investments in urban construction funds and basic public infrastructure, well-developed transportation facilities, and a positive trend in ecological environment development. The “Multi-Core” refers to the development model where Guiyang serves as the central core, driving the development of surrounding regions such as Anshun, Kaili, and Duyun, as well as adjacent counties; Zunyi acts as the northern core, stimulating economic growth in the northeastern part of Guizhou; Tongren functions as the eastern core, promoting the development of surrounding counties; and Liupanshui, as the western core, leads the development of Bijie and Xingyi.

The realization of this scenario necessitates a multifaceted approach. Firstly, leveraging the Shanghai-Kunming and Chongqing-Qianxiang High-Speed Railways to interconnect the core cities across eastern, central, and western Guizhou, thereby fostering urbanization along the routes and attracting capital influxes into these hubs. This, coupled with harnessing local distinctive industries such as Guiyang-Gui’an’s big data sector, Tongren’s ecological tea, traditional medicinal materials, edible mushrooms, and Liupanshui’s kiwifruits and prickly pears, will radiate economic benefits to surrounding areas. Secondly, augmenting funding for innovative research to upgrade and optimize energy consumption in big data storage, while fostering quality and packaging innovations for local specialty products. Thirdly, reinforcing the construction of basic public service facilities in counties and townships to enhance citizens’ well-being and foster human-centric urbanization. Fourthly, enhancing openness to the outside world to unleash Guiyang’s potential as a megacity, guiding the agglomeration of high-tech industries, and simultaneously elevating the development levels of neighboring cities and counties like Tongren, Xingyi, Liupanshui, and Bijie. Lastly, amidst regional urbanization efforts, emphasizing the development of nature reserves to safeguard biodiversity and prevent species loss. Nevertheless, the “multi-core” development approach can elicit competition among core cities, leading to uneven resource allocation and redundant construction. It becomes challenging to balance effective coordination, capital investment, and benefit distribution among core cities, as well as economic development and ecological protection.

③ The “One Body with Two Wings” development scenario represents a comprehensive framework based on maximal variations in influencing factors, featuring rapid escalation of innovation capabilities, substantial investments in urban construction funds, coordinated advancements in basic public service infrastructure, an extensively interconnected provincial transportation network, and a continually improving ecological environment. This scenario posits the formation of a “One Body” encompassing the regions of Guiyang, Duyun, Kaili, Zunyi, Bijie, Liupanshui, and Anshun, while Xingyi in southwestern Guizhou and Tongren in northeastern Guizhou serve as the “Two Wings.” Ultimately, this model envisions the rapid development of the “One Body” in central Guizhou as the core, with Xingyi and Tongren acting as central hubs for the western and eastern urban clusters, thereby fostering coordinated development across eastern, central, and western regions and realizing an organically unified urbanization spatial pattern for Guizhou Province.

The realization of this scenario necessitates a multi-faceted approach. Firstly, innovation must be placed at the forefront, as it serves as the primary driving force for development. Continuous investment in innovation should be prioritized to promote technological advancements and talent cultivation, thereby providing ample momentum for urbanization. Secondly, rational planning of regional industrial development is crucial, with both overall and detailed planning requiring implementation to drive the optimization of urbanization spatial patterns within designated zones. Thirdly, a robust foundation in infrastructure transportation is imperative, with the construction of north-south and east-west trunk lines and transportation hubs playing a pivotal role in integrating eastern and western Guizhou, laying the groundwork for urbanization. Fourthly, the provision of basic public services must be rational, scientific, and well-executed, fostering a willingness among the populace to migrate to urban areas and earning popular support for urbanization efforts. Lastly, tailored ecological construction and environmental governance schemes should be devised for distinct regions, bringing Guizhou closer to achieving its goal of becoming a “National Ecological Civilization Pilot Zone”. However, the implementation of the “One Body with Two Wings” development scenario will encounter numerous uncertainties, such as policy adjustments and market fluctuations, thereby increasing the difficulty of implementation. This development scenario exerts considerable pressure on resources and the environment and may further widen the development disparities among regions, particularly the gap between the two wing areas and the core area. Guizhou is relatively weak in terms of innovative resources and talent pools. How to attract and cultivate high-level talents in the context of the “One Body with Two Wings” development scenario poses a significant challenge.

### Scenario evaluation and selection

#### Scenario assessment.

The subjective weighting method leverages experts’ theoretical knowledge and practical experience to assign reasonable weights to evaluation indicators, and can effectively determine the ranking of each indicator based on its importance. This approach avoids the potential inconsistency between indicator weighting and reality that may arise from purely objective weighting methods. Thus, based on the developmental scenarios and the current state of Guizhou Province, this study employs the Analytic Hierarchy Process (AHP) to construct an evaluation index system for strategic selection of urbanization development patterns under the overarching goals of optimizing the spatial pattern of urbanization in Guizhou and fostering regional coordination. This system encompasses various indices, with their respective weights being calculated at each level (Table 4). These weights serve as a guide for strategic decision-making in urbanization development. The determination of the weights for indicators at all levels is based on the experience of decision-makers or experts.

**Table 4:** Evaluation Index System for Strategic Selection of Urbanization Development Patterns.

Primary indicator	Secondary indicator
Innovation ability(0.15)	Research innovation (0.40): innovation in the output capacity of science and technology
	Educational innovation (0.35): the cultivation of students’ creativity and the innovation of educational activities
	Innovation sustainability (0.25): Sustainability of investment in innovation projects
Spatial layout(0.35)	Layout rationality (0.30): layout rationality of cities of different sizes
	Industrial coordination (0.35): The degree of coordinated development among industries
	Rural-urban closeness (0.15): communication closeness between urban and rural areas with different functions
	Ease of living (0.20): the ease with which residents of different occupations live and work
Infrastructure(0.2)	Educational universality (0.30): The prevalence of children being fully educated in different regions
	Medical accessibility (0.30): Residents of all economic levels have access to good medical care
	Transport accessibility (0.25): The accessibility of transport links between different regions
	Transportation unity (0.15): Multiple modes of transportation operate in harmony
Fund Supply (0.2)	Diversity of funds (0.15): Diversity of sources of funds
	Capital adequacy (0.30): the adequacy of funds to ensure urban and rural construction
	Sustainability of funding (0.35): Sustainability of funding provision
	Capital rationality (0.20): The reasonable degree of capital investment required in different areas
Ecological Environment (0.1)	Urban and rural ecology (0.30): The degree to which urban and rural residents live in harmony with nature
	Environmental quality (0.30): The good development of ecological environment in different regions
	Policy orientation (0.25): favorable policies proposed by the government for ecological environment development
	Land use intensity (0.15): intensity of land use

In the single-core development scenario, resources are overly concentrated, leading to relatively weak innovation capacity and spatial layout, although centralized investment and construction result in well-developed infrastructure. The flow of funds and financing channels in single-core development may present certain issues, hence the funding supply in this scenario should be considered at a moderate level. The inherent ecological environment is favorable, but attention should still be paid to the impact of single-core development on the ecological environment. In the multi-core development scenario, interaction and competition among regions are conducive to stimulating innovation capacity, enhancing infrastructure utilization efficiency and service levels, and achieving balanced resource distribution and urban development, thereby making the urban spatial layout more reasonable. Multi-core development faces challenges in fund allocation and coordination, but the funding situation is still better than that of the single-core scenario. Multi-core development can disperse environmental pressure and achieve regional sustainable development. In the “One Body with Two Wings” development scenario, collaborative innovation and resource sharing contribute to enhancing overall innovation levels and the overall efficiency of infrastructure, which further aids in resource optimization and balanced urban development. The “One Body with Two Wings” development faces more complex challenges in fund coordination and management, but the funding situation remains the best among the three scenarios. However, the rapid development of the “One Body with Two Wings” model will inevitably have a certain impact on the ecological environment. Using the Fuzzy Comprehensive Evaluation Method, this analysis evaluates the strategies and implementation pathways for the three urbanization development scenarios: “single-core,” “multi-core,” and “One Body with Two Wings.” The evaluation values are represented by the degree of membership of each indicator to the category “excellent” (Table 5), with higher values indicating better performance.

**Table 5:** Results of fuzzy comprehensive evaluation.

Development scenario	Innovation ability	Spatial layout	Infrastructure	Fund Supply	Ecological Environment	Composite value
Mononuclear	0.26	0.29	0.46	0.34	0.47	0.35
Multinuclear	0.43	0.50	0.56	0.40	0.60	0.49
One body with two wings	0.59	0.72	0.76	0.59	0.56	0.67

As evident from the evaluation results of individual sub-objectives, the “multi-core” development scenario exhibits the highest degree of membership to “excellent” in terms of ecological environment, surpassing the other two scenarios. However, the “One Body with Two Wings” scenario significantly outperforms the other two in the remaining four dimensions of indicators and achieves the highest overall score. Therefore, among the three development scenarios, the “One Body with Two Wings” scenario is deemed more ideal, aligning better with the long-term development trends of Guizhou. It is also more supportive of Guizhou’s strategic development goals as a “National Ecological Civilization Pilot Zone,” “National Big Data Comprehensive Pilot Zone,” “Inland Open Economy Pilot Zone,” and “Western Rural Revitalization Pilot Zone.”

#### Scenario selection.

Through the analysis and evaluation of different scenarios, it is found that all constructed urbanization spatial pattern scenarios have the potential for realization. However, the “One Body with Two Wings” scenario stands out in its ability to concentrate advantages on the development of urban clusters centered around Guiyang, Zunyi, Liupanshui, and their peripheries, leveraging the polarizing and driving effect of Guiyang as a megacity. Simultaneously, it fosters the creation of two new growth poles in Zunyi and Liupanshui, as well as promoting cluster development in Xingyi and its surroundings in southwestern Guizhou, and in the Tongren vicinity in northeastern Guizhou. This scenario prioritizes coordinated development of urban clusters in western and eastern regions, strengthening transportation networks and economic ties across the province, and balancing development progress across regions. Consequently, this paper advocates adhering to a spatial development strategy of “strengthening the central region, forming urban clusters, establishing robust fulcrums, and fostering coordinated development,” thereby constructing an urbanization spatial development pattern of “One Body with Two Wings.”

**Fig 6:**
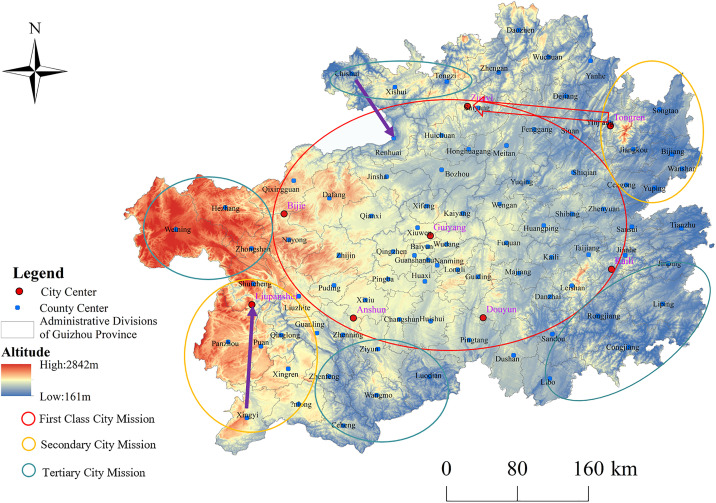
“One body and two wings” development diagram. Note: This map was created based on the standard map with approval number GS(2024)0650 downloaded from the Map World Service Center (https://cloudcenter.tianditu.gov.cn/dataSource), of the National Geographic Information Public Service Platform, and no modifications were made to the base map. When reprinting or quoting this content, it is necessary to clearly indicate “Reprinted from (or cited from) Tiandi Map (https://www.tianditu.gov.cn)”.

The development of an urbanization spatial pattern of “One Body with Two Wings” involves establishing Guiyang as the primary core and Zunyi, Liupanshui, Bijie, Anshun, Duyun, and Kaili as supporting nodes within the main development zone of Guizhou. This also entails forming urban clusters in southwestern Guizhou centered on Xingyi and in northeastern Guizhou centered on Tongren. By leveraging and expanding transportation advantages, Zunyi can lead Tongren, while Liupanshui guides Xingyi, and Renhuai collaborates with Chishui. By enhancing the radiating capacity, establishing effective interest coordination mechanisms and market mechanisms, as well as leveraging the driving effects of major cities, this strategy aims to promote coordinated regional development and nurture vibrant and potential regional growth poles. Additionally, implementing effective policies to accelerate the construction of small- and medium-sized cities and towns, optimizing urban structures, will ultimately contribute to the formation of an urbanization spatial pattern that promotes regional coordination (Fig 6).

## Discussion

### Urbanization spatial pattern evolution

The evolution of urbanization spatial patterns constitutes a complex, multidimensional process characterized by dynamic interactions among various socioeconomic factors. Our analysis reveals three key dimensions of this evolutionary process in Guizhou Province: firstly, the spatio-temporal trajectories of core development elements demonstrate a clear concentration trend. The economic, demographic, and employment centers all exhibit gravitational movement toward Guiyang’s urban core, confirming its role as the province’s primary growth pole. This spatial convergence not only reinforces Guiyang’s dominant position through significant polarization effects but also accelerates provincial urbanization through spillover mechanisms. Secondly, regional development patterns reveal marked spatial disparities. Central Guizhou emerges as the socioeconomic pillar, with its population density, economic output, and per capita GDP substantially exceeding those of eastern and western regions. The analysis further indicates that metropolitan districts serve as critical development engines, driving overall provincial progress through their concentrated resources and administrative functions. Thirdly, the evolutionary dynamics show increasing spatial-economic integration. The nearly parallel migration paths of economic and population centers, coupled with the gradual alignment of employment distribution, reflect growing coordination between economic development, population agglomeration, and labor market evolution in Guizhou. Meanwhile, Guiyang’s supportive role as a regional hub remains prominent, with central Guizhou continuing to function as the developmental backbone. The continuous improvement of transportation networks has played a particularly crucial role in optimizing the spatial configuration, facilitating interregional connectivity and resource mobility.These findings collectively validate the persistent spatial imbalance in Guizhou’s urbanization pattern while highlighting the central region’s pivotal position in provincial development. The results provide empirical evidence for understanding the complex mechanisms underlying urbanization spatial evolution in mountainous regions.

### Factors influencing urbanization spatial patterns

Beyond the three core elements of economic output, population size, and employment conditions, numerous other factors play significant roles in promoting regional coordinated development and rational urbanization spatial distribution. Firstly, innovation serves as the primary driver of development. Guizhou Province adheres to an innovation-driven development strategy, fostering an environment conducive to technological and talent support, which is essential for achieving high-quality urbanization. Secondly, investment in urban construction funds directly impacts urban development, scale, and construction quality. Such investment facilitates the realization of regional planning objectives, promotes the implementation of large-scale projects, and generates employment opportunities and economic benefits. The diversification of funding sources and rational allocation of resources further enhance these effects. Similarly, the development of public services is a critical component of urbanization, addressing the basic needs of rural migrants and strengthening their sense of belonging. High-speed rail networks, exemplified by the Shanghai-Kunming High-Speed Railway, have improved the mobility of resources and labor within the region, laying the foundation for economic growth. The government has made relentless efforts in road construction, striving to establish a four-tier transportation network connecting provincial, municipal, county, and town levels. This infrastructure is vital for optimizing urbanization spatial patterns and fostering regional coordination. Finally, ecological and environmental changes present both challenges and opportunities. The pursuit of economic benefits has sometimes overlooked ecological constraints, leading to environmental degradation. However, these challenges also point toward a path of sustainable development, emphasizing harmonious coexistence between humans and nature. Urbanization must be grounded in ecological security, charting a new course distinct from past approaches to promote regional coordinated development.In summary, these factors interact dynamically, collectively shaping the evolution of urbanization spatial patterns in Guizhou Province.

### Scenario analysis and evaluation of urbanization spatial patterns

Through scenario-based simulation and comprehensive evaluation, this study systematically demonstrates the optimal suitability of the “One Core with Two Wings” strategy in Guizhou’s urbanization process. The proposed model establishes an integrated development framework centered on the Guiyang-Zunyi-Liupanshui growth pole, which effectively coordinates urban clusters in eastern and western regions through a “point-axis” spatial system. This configuration achieves dual objectives: enhancing the radiating capacity of central regions while promoting balanced regional development via optimized transportation networks. Compared to the “single-core” model’s resource siphoning effect and the “multi-core” model’s competitive internal friction, the “one body, two wings” strategy demonstrates significant advantages in innovation synergy, resource optimization, and ecological carrying capacity, securing the highest overall score. While facing challenges such as complex financial coordination and ecological pressures, its functional zoning and industrial complementarity mechanisms effectively enhance overall efficiency. Recommendations include strengthening strategic resilience through cross-regional interest coordination, institutional innovation in ecological compensation, and digital planning tools. Moving forward, it is crucial to establish dynamic monitoring mechanisms to ensure effective alignment between policy responses and market changes, thereby continuously unlocking the multiplier effect of the “one body, two wings” framework.

## Conclusion

A scientifically rational urbanization spatial pattern serves as the driving force for urbanization development and a pivotal strategy in advancing regional coordinated development, holding significant implications for high-quality urbanization. Taking Guizhou Province, China, as a case study, this paper, grounded in the evolutionary characteristics and existing issues of Guizhou’s core elements, applies scenario analysis to the optimization of urbanization spatial patterns, integrating qualitative and quantitative approaches to analyze this optimization from both theoretical and practical perspectives. The main conclusions are as follows:

① Leveraging “optimization of urbanization spatial patterns” as the primary lever, with promoting regional coordinated development as the core objective, and based on scenario analysis, an optimization pathway of “urbanization spatio-temporal evolution - summary of pain points and difficulties - scenario simulation setup and optimization - strategic goal selection” is constructed and applied to the optimization practice in Guizhou Province.
② The key features and related issues in the evolution of Guizhou’s urbanization spatial pattern are analyzed. Key features include coordinated development of regional centers and the prominent supporting role of Guiyang as a megacity. Major issues manifest in the incongruence between employment levels and economic development, excessive disparities in city sizes leading to weak overall competitiveness and unbalanced development, intensified polarization trends in central Guizhou, and inadequate transportation infrastructure with limited capacity to drive economic growth.
③ The primary influencing factors are identified as economic aggregate, population size, employment status, innovation drive capacity, urban construction funding, public service development, transportation infrastructure construction, and ecological environment changes.
④ Through scenario construction, analysis, and evaluation, a spatial development strategy and orientation of “strengthening central Guizhou, fostering urban clustering, solidifying development fulcrums, and promoting regional coordinated development” is proposed, thereby constructing a “One Body with Two Wings” urbanization spatial development pattern for Guizhou.

Currently, research on urbanization spatial patterns remains insufficiently in-depth, lacking a comprehensive framework encompassing optimization logical pathways and analytical methodologies (He et al., 2016; Turner & Kaplan, 2019) [29,30]. Drawing upon scenario analysis and integrating the characteristics of urbanization spatial patterns derived from gravity center analysis, this paper proposes optimization scenarios for the development of urbanization spatial patterns. Applying these research findings to the optimization of urban patterns in Guizhou Province to facilitate regional coordinated development can assist in addressing uncertainties in urbanization spatial patterns and human initiative. However, scenario analysis possesses blind spots, as it may struggle to cope with complex developmental trends, both logically and in terms of coordination (Liebl, 2002) [31]. Some scholars argue that the logic of development scenarios may potentially hinder the derivation of correct conclusions (Lempert et al., 2003) [32]. It is challenging to comprehensively capture all variables and their interactions that influence the spatial pattern of urbanization, especially in the context of rapidly changing socio-economic environments. Secondly, scenario building often relies on expert judgments and assumptions, which may lead to subjectivity and uncertainty in analysis results. Furthermore, there are differences in the spatial patterns of urbanization across different regions, and adjustments should be made based on their respective natural, economic, and social conditions when studying other regions to ensure the adaptability and operability of optimization strategies. Future research directions on the spatial pattern of urbanization can integrate advanced technologies such as big data and artificial intelligence to improve the accuracy and efficiency of scenario analysis. Additionally, interdisciplinary research methods can be employed to provide new perspectives and tools for optimizing the spatial pattern of urbanization. Therefore, scenario analysis necessitates integration with a diverse range of methods. Additionally, the scenario forecasting methods and optimization pathways proposed in this paper await practical validation in relevant regions to achieve the intended goals.

## Supporting information

S1 Data Data. (XLS)
